# Reliable ligamentous stability and high return-to-sport rates after arthroscopic reduction and internal fixation of tibial eminence fractures

**DOI:** 10.1007/s00402-021-03961-6

**Published:** 2021-05-19

**Authors:** Patricia M. Lutz, Stephanie Geyer, Philipp W. Winkler, Markus Irger, Daniel P. Berthold, Matthias J. Feucht, Andreas B. Imhoff, Philipp Forkel

**Affiliations:** 1 Department for Orthopedic Sports Medicine, Technical University Munich, Ismaninger Str. 22, 81675 Munich, Germany; 2 Department of Orthopedics and Trauma Surgery, Medical Center, Faculty of Medicine, Albert-Ludwigs-University of Freiburg, Freiburg, Germany

**Keywords:** Tibial eminence fracture, Tibial spine, Adolescent ACL, ARIF, Suture fixation, Return to sport

## Abstract

**Purpose:**

To investigate functional and clinical outcomes, and physical activity after arthroscopic suture fixation of tibial eminence fractures with regard to postoperative stability, range of motion (ROM), complications, and return to sports.

**Methods:**

Patients undergoing arthroscopic reduction and internal fixation (ARIF) of tibial eminence fractures using a suture fixation technique were included. Outcome was evaluated retrospectively after a minimum follow-up of 24 months using KT-1000 arthrometer measurements, clinical examination, outcome scores (Lysholm score, Tegner Activity Scale), and a questionnaire about sport activities.

**Results:**

A total of 23 patients (44% male, 57% female) with a mean age of 25 ± 15 years were included. Mean follow-up was 57 ± 25 months. KT-1000 arthrometer measurements of anterior tibial translation revealed a mean side-to-side difference of 0.9 ± 1.0 mm. Clinical examination showed 100% normal or nearly normal anterior translation of the tibia. Two patients (9%) received an ACL reconstruction due to traumatic ACL re-instability and were, therefore, considered as failures. An extension deficit concerning hyperextension occurred in 29% of patients postoperatively. Further postoperative complications occurred in 14% of patients and included postoperative stiffness with ROM limitations and secondary dislocation of a fragment. Mean postoperative Lysholm score was 89 ± 14. Comparing pre- and postoperative values, no significant change of the Tegner Activity Scale was observed. All patients (failures excluded) returned to high impact sports activities after ARIF.

**Conclusion:**

Excellent reliable ligamentous stability and high rates of return to high impact sports can be expected after ARIF using a suture fixation technique for type II–IV tibial eminence fractures. Complications, such as limitations in ROM, commonly occur in up to 30% after ARIF. Therefore, regular follow-up examinations remain important in this usually young patient cohort.

**Level of Evidence:**

Level IV.

## Introduction

Avulsion fractures of the tibial eminence and subsequent acute anterior cruciate ligament (ACL) deficiency are often referred as a rare injury in the adult population as they mostly occur in children and adolescents due to their skeletal immaturity, muscle weakness, and increased elasticity of the ligaments [26, 27]. However, these injuries can generally affect all age groups and show a high association with concomitant meniscal injuries [6, 8, 10, 27]. First described by Meyers and McKeever in 1959, patients with instable type II–IV injuries [24, 25, 46] are generally indicated for surgical stabilization, regardless of their age [5, 6, 10, 17, 40]. Current literature covers several open and arthroscopic surgical approaches of internal reduction and fixation, mainly including screws or high-tensile suture devices with postoperative arthrofibrosis and secondary ACL deficiency requiring reconstruction being the most common complications [2, 4–6, 10, 17, 23, 26, 31, 32, 36, 44, 45]. Recently, satisfactory patient-reported and functional outcomes in the treatment of intercondylar eminence fractures in children and adolescents with bio-absorbable nails were reported [19]. Generally, good clinical and functional outcomes have been reported after arthroscopic reduction and internal fixation (ARIF) of tibial eminence avulsion fractures, however, these studies are often limited to their small sample size [3, 5, 6, 10, 11, 17, 18, 30, 31, 33, 36, 44]. Although a vast number of studies investigated postoperative outcomes and complications following ARIF, there remains a lack of data reporting on postoperative ligamentous stability, return to sports, and physical activity. However, this may be of clinical relevance, as this younger patient cohort in particular, often comes along with high expectations concerning postoperative level of return to sports and physical activity.

Therefore, the purpose of this retrospective study was to investigate clinical and functional outcomes, return to sports, and physical activity after ARIF of tibial eminence avulsion fractures using high-tensile suture devices in patients suffering from instable grade II–IV tibial eminence fractures. It was hypothesized that the mean difference in anterior tibial translation between the affected, injured and non-affected, healthy leg is smaller than 3 mm (mm) measured using a validated KT-1000 arthrometer.

## Methods

The study was approved by the institutional review board of the Technical University of Munich (341/20 S). All patients and their parents, if under 18-years old, gave their written informed consent to participate in this investigation.

### Patients

**Fig. 1:**
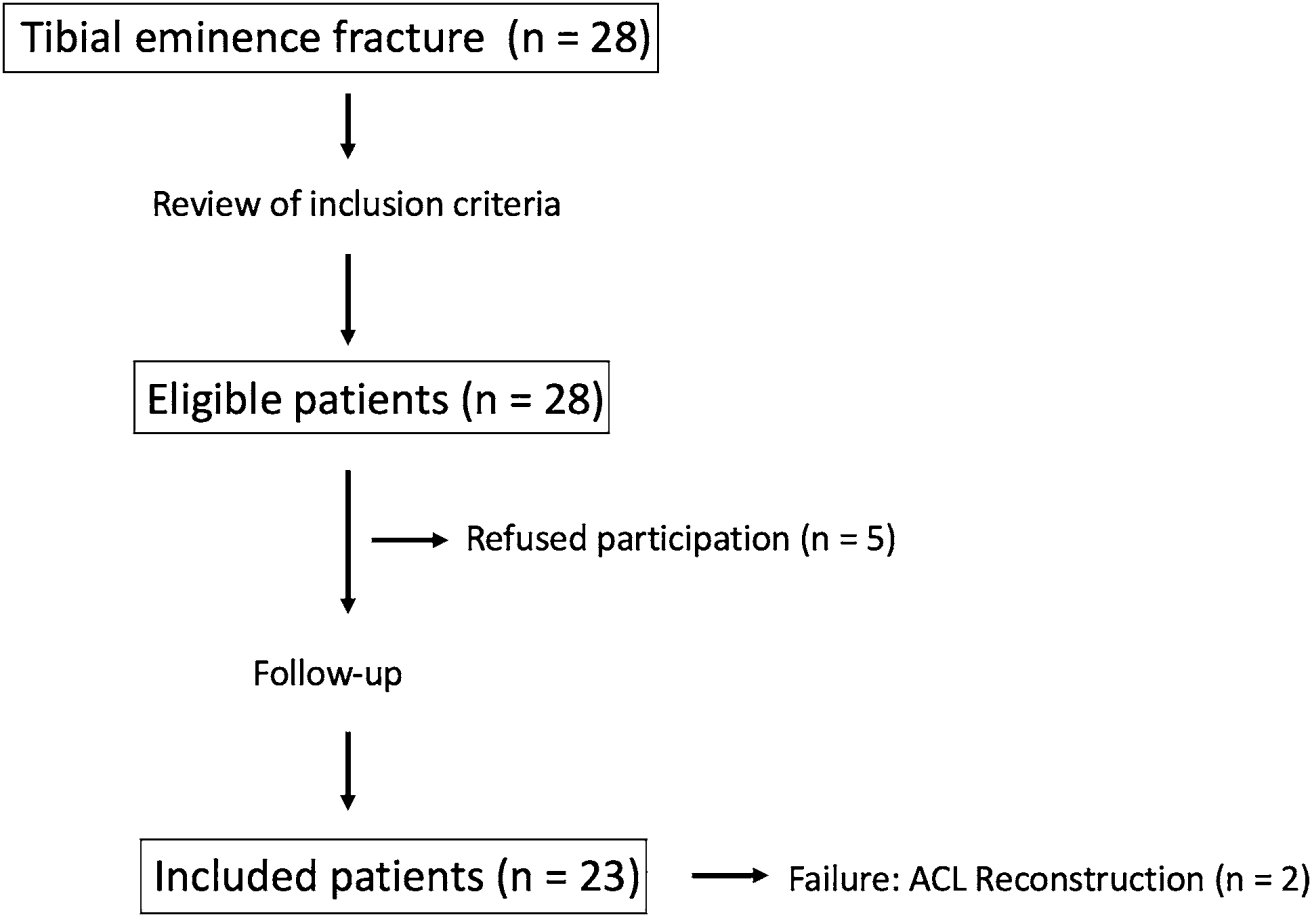
Enrollment flowchart of the study. *ACL *anterior cruciate ligament

A retrospective chart review was performed on a consecutive cohort of all patients undergoing ARIF after tibial eminence avulsion fractures at the authors’ institution between July 2011 and July 2018. Patients were eligible for inclusion if noted to have unilateral ARIF following type II–IV tibial eminence avulsion fractures according to Meyers and McKeever [25] modified by Zaricznyj [46] with or without concomitant meniscus lesions as initially noted on magnet resonance imaging (MRI) and confirmed arthroscopically at the time of surgery. The Meyers and McKeever classification describes four subtypes, with type 1 being non-displaced and type 4 being a displaced multi-fragment avulsion of the tibial eminence [25, 46]. Patients were excluded from the study if they had a history of previous surgery at the index or the contralateral, non-affected knee, and/or concomitant ligamentous injuries other than avulsion fractures of the ACL. Baseline demographic variables, including patient age and gender, were manually collected using clinical notes of all patients. Failure was defined as secondary ACL deficiency requiring reconstruction (ACL-R) after ARIF. The patient selection process is shown in Fig. 1.

### Radiological assessment

Prior to surgery, all patients had undergone a thorough clinical and radiological (X-rays, MRI, and, if indicated, computed tomography (CT) scans) examination to detect tibial eminence avulsion fractures with or without concomitant meniscus lesions. To confirm the correct position of the suture and complete reduction of the fragment, standardized anterior–posterior (a.p.) and lateral radiographs were carried out postoperatively.

### Clinical examination

All subjects underwent postoperative clinical examination of the knee, including previously validated KT-1000 arthrometer measurements (MEDmetric, San Diego, CA, USA) [1, 7, 9, 15, 34, 45], by a knee trained orthopedic resident (P.M.L) at a minimum follow-up of 2 years. Clinical evaluation of the patient’s knee joint was based on the International Knee Documentation Committee (IKDC) knee examination form [12, 13]. Free range-of-motion (ROM) was measured using a goniometer. Clinical examination of the ligament stability included testing the laxity of the anterior and posterior cruciate ligament, as well as the medial collateral ligament (MCL) and lateral collateral ligament (LCL). Anterior tibial displacement was first evaluated using the Lachman test and quantitatively measured using the KT-1000 arthrometer. The KT-1000 arthrometer measurements were performed using a standardized 134 N anterior draw force at 30° knee flexion and side-to-side differences were recorded in mm. The KT-1000 arthrometer measurements were performed using the maximal manual forces and side-to-side differences were recorded in mm. The pivot-shift test was graded as negative, 1 + (glide), 2 + (clunk), or 3 + (locking) [14, 41]. All results were compared with the non-affected knee and documented. Any meniscus pathology was evaluated by joint space tenderness, the Steinmann test [39] and as initially noted on MRI and confirmed arthroscopically at time of surgery.

### Functional outcomes

Patient-reported outcomes were measured using the Lysholm score and the Tegner Activity Scale at final follow-up [12, 42]. Similar to Xu et al. and Dung et al. a differentiation of the Lysholm score results into excellent (95–100), good (85–94), fair (65–84), and poor (< 65) was made [7, 45]. To provide a concise understanding of the sport activity level in this collective, patients were asked about pre- and postoperative types of sport activities, activity levels, and return-to-sport activities. The subjective postoperative sports ability could be rated on an ordinal scale (“improved”, “equal to preoperative state”, or “deteriorated”) and reasons for a subjective deterioration in physical activity were investigated (“due to the operated knee”, “due to other physical problems not related to the operated knee”, or “due to non-physical personal reasons”).

### Surgical technique

All surgical procedures were performed by fellowship-trained senior orthopedic surgeons (P.F. and A.B.I).

**Fig. 2:**
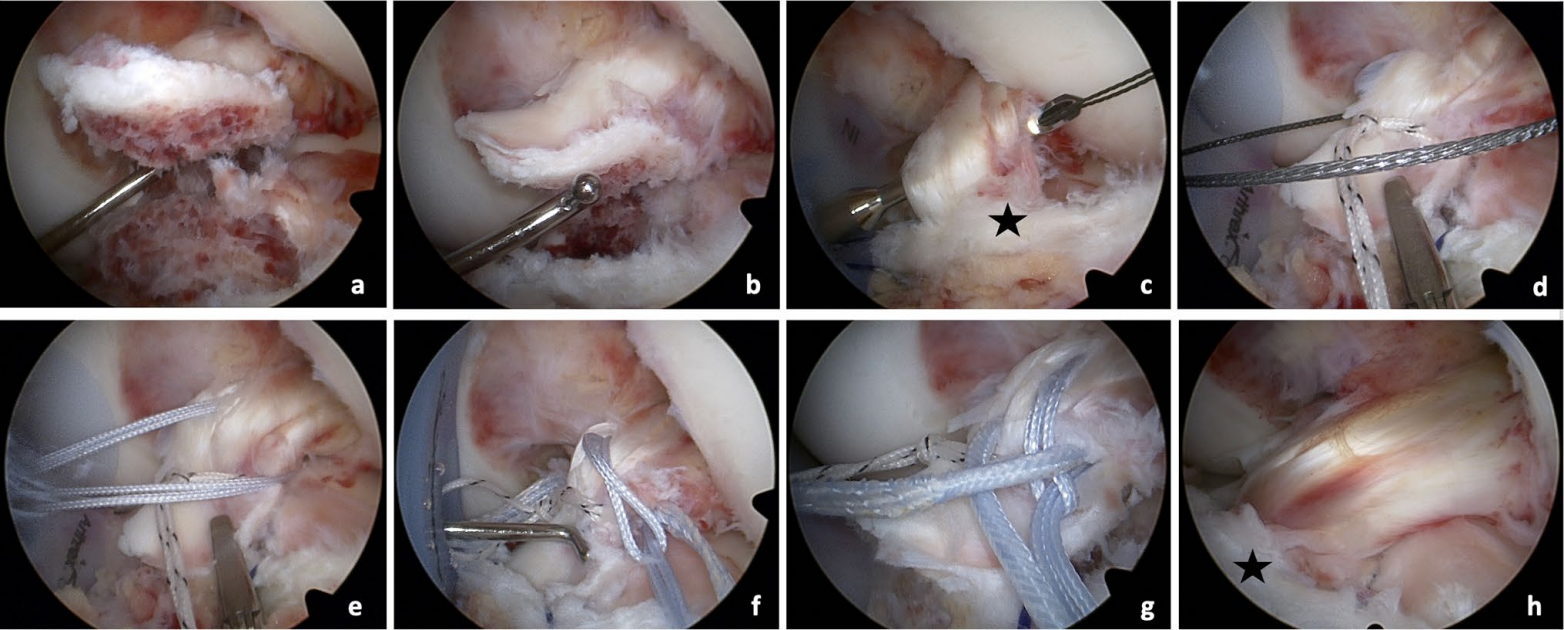
ARIF of tibial eminence fracture. **a** confirmation of tibial eminence fracture; **b** exposure of the instable fractured tibial eminence fragment; **c** a SutureLasso (Arthrex Inc., Naples, FL, USA) is passed through the distal ACL from posteromedial in an anterolateral direction; **d** a FiberWire Number 2 (Arthrex Inc., Naples, FL, USA) is shuttled through the ACL; **e** a second suture is shuttled through the anterior part of ACL; **f**–**g**, ARIF of the fractured tibial eminence; **h**, reposition of the fractured fragment with an individual adaptation of tension and stability of the native ACL; *Black star marks transverse ligament. ACL *anterior cruciate ligament, *ARIF* arthroscopic reduction and internal fixation

**Fig. 3:**
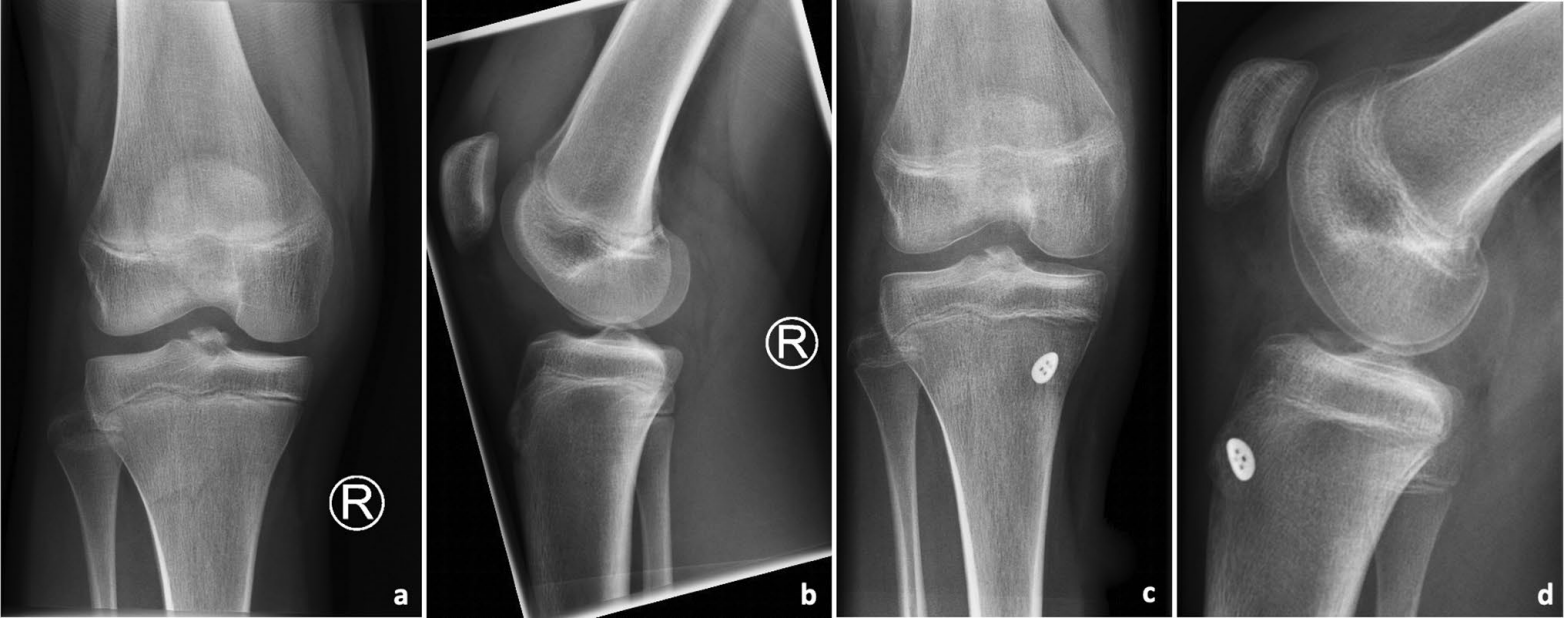
Standardized anterior–posterior and lateral radiographs in a right knee showing a tibial eminence avulsion fracture preoperatively (**a**–**b**) and on the first postoperative day after ARIF (**c**–**d**). For tibial fixation, an extracortical suture plate was used (**c**–**d**). *ARIF *arthroscopic reduction and internal fixation

In all patients, ARIF of the avulsion fracture was performed (Fig. 2). Routine diagnostic arthroscopy was performed through a high anterolateral portal. In case of any concomitant meniscus lesions, meniscus suture systems (Arthrex Inc, Naples, FL, USA) were used or partial resection of the meniscus was performed prior to ARIF if indicated. Subsequently, the instable fractured tibial eminence fragment was exposed and carefully debrided. The transverse ligament was visualized and the tibial fracture site was debrided. Then a curved 90° SutureLasso (Arthrex Inc., Naples, FL, USA) was passed through the distal ACL from posteromedial in an anterolateral direction through an anteromedial portal. A FiberWire Number 2 (Arthrex Inc, Naples, FL, USA) was shuttled through the ACL. A second suture was shuttled through the anterior part of ACL. Two transtibial K-wires were then placed mid into the instable eminence fragment to securely fix the fragment, and were then subsequently overdrilled. Then, the suture was shuttled through the drill holes and by pulling on the free ends of the FiberWire sutures (Arthrex Inc, Naples, FL, USA), the fractured eminence fragment was then securely repositioned into the fracture site. Catching of the transverse ligament was avoided. For tibial fixation, an extracortical suture plate was used. To confirm the correct position of the suture and complete reduction of the fragment, standardized a.p. and lateral radiographs were carried out intra-operatively (Fig. 3).

### Rehabilitation

The postoperative protocol consisted of 6 weeks of partial weight-bearing (20 kg) on crutches with limitations to active and passive ROM (Extension/Flexion 0°/0°/90°) [32]. A knee brace (Medi M4, Medi Bayreuth, Germany) was provided for at least 12 weeks. When performing additional meniscal repair, ROM was restricted from 0° to 60° of knee flexion for the first 6 weeks to protect the construct. Return-to-sport-specific training was allowed after 3 months and full return to contact and/or pivoting sports activities after at least 6 months postoperatively.

### Statistical analysis

All statistical analyses were performed using SPSS software Version 25 (IBM, Armonk, New York, USA) and Microsoft Excel Version 2019 (Microsoft, Redmond, Washington, USA). For all statistical tests, p values less than 0.05 were considered as statistically significant. Kolmogorov–Smirnov univariate normality test was used for continuous variables to confirm data normality. Descriptive statistics were presented as mean ± standard deviation (SD) for all continuous variables. Frequencies (n, %) were used to obtain descriptive statistics for all categorical variables. The one-sample t test was applied to test for the null hypothesis (true mean difference equals 3 mm). The Wilcoxon test was conducted to test for differences in Tegner Activity Score between pre-operative and postoperative measurements.

In the study of Pandey et al. [31], a mean side-to-side difference of 0.85 mm and a standard deviation (SD) of 0.90 mm were observed in KT-1000 measurements. Even under a conservative assumption of a mean difference of 1.5 mm and a SD of 1.5 mm, with a sample size of 21 patients (failures excluded), the power analysis revealed a power of 90% to reject the null hypothesis in a one-sample t test (significance level of 5%, two-sided test).

## Results

In total, 28 patients underwent ARIF of tibial eminence avulsion fractures in the authors’ institution between July 2011 and July 2018. Of these 28 patients, two patients received an ACL-R due to traumatic ACL re-instability and were, therefore, considered as failures; Five patients were not willing to participate, leaving 21 patients examined in the present study. Patient demographics of the total study group are shown in Table 1. Concomitant meniscal injuries were found in eight patients (35%).

**Table 1:** Patient demographics of the total study group

Number of patients, *n*	23
Follow-up (months)	57.1 ± 24.7 (28–103)
Age (years) at surgery	24.5 ± 15.4 (6–52)
Sex, *n* (%)	
Male	10 (43.5%)
Female	13 (56.5%)
BMI (kg/m^2^)	23.0 ± 4.7 (16.0–37.6)
Laterality, *n* (%)	
Right	10 (43.5%)
Left	13 (56.5%)
MM type, *n* (%)	
Type II	10 (43.5%)
Type III	12 (52.2%)
Type IV	1 (4.3%)
Mechanism of injury, *n* (%)	
Skiing accident	15 (65.2%)
Hoverboard accident	2 (8.7%)
Bike accident	2 (8.7%)
Sports accident (basketball, soccer)	4 (17.4%)
Concomitant procedures, *n* (%)	
None	15 (65.2%)
Partial resection of meniscus	1 (4.3%)
Meniscal repair	7 (30.4%)

Continuous variables are shown as mean ± standard deviation (range), categorical variables are shown as percentages

*BMI *body mass index,* MM type *modified Meyers and McKeever classification type

### Clinical presentation and outcome

After a mean follow-up of 57 ± 25 months (28–103), clinical examination showed a 1 + Lachman in two patients and a 1 + pivot-shift test in three patients. ROM measurement using the goniometer showed an extension deficit in six patients concerning hyperextension. No flexion deficit occurred in the study group. Mean side-to-side difference for anterior translation of the tibia, measured with the KT-1000 arthrometer, was 0.9 ± 1.0 mm (95% confidence interval (CI) 0.4–1.4; *p* < 0.001). Results of clinical examination are summarized in Table 2.

**Table 2:** Clinical results of the examined study group

Clinical results	Study group*
*KT-1000, side-to-side difference* (*mm)*	0.9 ± 1.0 ( – 1.6 to 2.3)
*Lachman test, n (%)*	
Negative	19 (90.5%)
1 +	2 (9.5%)
*Pivot-shift test, n (%)*	
Negative	18 (85.7%)
1 +	3 (14.3%)

Continuous variables are shown as mean ± standard deviation (range), categorical variables are shown as percentages. *n = 21 patients;

*KT-1000 *manual maximum displacement test,* SD *standard deviation,* mm millimeter*

### Lysholm score and return-to-sports

The descriptive data for postoperative Lysholm score, pre-operative and postoperative Tegner Activity Scale, return-to-sport activities, and physical activity are summarized in Table 3. At final follow-up, 100% of patients were involved in sport activities. In general, frequency of sport activities was 2.5 ± 1.5 sessions per week preoperatively and 2.4 ± 1.7 sessions per week postoperatively (n.s.). In the patient subgroup that reported a reduced frequency of sports activity postoperatively, two patients (10%) attributed the deterioration to the operated knee, while five patients (24%) accredited it to non-physical personal reasons.

**Table 3:** Physical activity and clinical outcome scores of the examined study group

	Study group*
Lysholm score	88.9 ± 14.0 (58–100)
Excellent, *n* (%)	12 (57.1%)
Good, *n* (%)	3 (14.3%)
Fair, *n* (%)	5 (23.8%)
Poor, *n* (%)	1 (4.8%)
TEGNER preoperative TEGNER postoperative	6^+^ (range, 1–10)^§^ 6^+^ (range, 1–10)^§^
Return-to-sport activities, *n* (%)	
Yes	21 (100%)
Change in postoperative sports ability	
No change, *n* (%)	15 (71.4%)
Improved, *n* (%)	4 (19.0%)
Deteriorated, *n* (%)	2 (9.5%)

Continuous variables are shown as mean ± standard deviation (range), categorical variables are shown as percentages. *n = 21 patients*;* ^+^ Values are median; ^§^ no significant difference between pre- and postoperative TEGNER* (p* = *0.102);*

*TEGNER* tegner activity scale

Postoperatively, the most commonly performed sport activities were cycling (81%), swimming (67%), skiing (67%), hiking (52%), and fitness (29%) (Table 4).

**Table 4:** Details of sport activity after ARIF of tibial eminence fractures

Type of sport	Study group*	
	Preinjury	Follow-up
Skiing	17 (81%)	14 (67%)
Cycling	15 (71%)	17 (81%)
Swimming	13 (62%)	14 (67%)
Hiking	12 (57%)	11 (52%)
Jogging	6 (29%)	4 (19%)
Soccer	6 (29%)	5 (24%)
Fitness	5 (24%)	6 (29%)
Tennis	3 (14%)	4 (19%)
Ice Hockey	2 (10%)	2 (10%)
Cross-country skiing	2 (10%)	3 (14%)
Dancing	2 (10%)	1 (5%)

Values are expressed as number of patients (percentage of patients) who performed the sport activities before the injury and at follow-up. Multiple answers were possible. *n = 21 patients

*ARIF *arthroscopic reduction and internal fixation

### Complications

Two patients suffered from postoperative stiffness with ROM limitations due to postoperative arthrofibrosis. A second look arthroscopy with subsequent arthrolysis was necessary to regain full ROM after 5 and 10 months, respectively. One patient suffered from secondary dislocation of the fractured fragment and removal of the fragment was necessary 5 days after the initial operation.

### Failures

Two younger patients (one male, one female) with a mean age of 13 ± 1 years (12–14), suffered from traumatic ACL re-instability after ARIF and required ACL-R. The causing mechanisms of re-injury were skiing and bike accidents.

## Discussion

The most important finding of the present study was that there were no side-to-side differences in the KT-1000 measurements between the injured and non-injured leg in patients after ARIF of a tibial eminence avulsion fracture after a mean follow-up of 57 ± 25 months. In addition, those findings were confirmed by the clinical examinations showing similar results with 100% Lachman negative or 1 + when manually tested. As such, a 100% return-to-sport activities were achieved with no change in postoperative Tegner Activity Scale and no significant reduction of postoperative frequency of sport activities was observed after isolated ARIF of tibial eminence fractures. Of interest, most tibial eminence avulsion fractures were noted to occur due to high-energy pivoting sport activities.

To date, only few authors assessed ligamentous stability using the KT-1000 after ARIF of tibial eminence avulsion fractures, which are mostly limited to small patient cohorts [3, 7, 11, 17, 31, 33, 45]. Generally, a side-to-side difference less than 3 mm indicates that native ligamentous stability may be restored [1, 38]. Similar to the results of 26 patients after an all-arthroscopic suture pull-out fixation of displaced tibial spine avulsion fractures reported by Pandey et al.[31], small side-to-side differences could be found in the present study (0.9 ± 0.9 versus 0.9 ± 1.0). Interestingly, Seon et al. used instrumented stress radiography to report an anterior tibial translation of under 5 mm in over 90% of their patients after ARIF in suture technique [36]. Coyle et al. described patients after tibial eminence fractures having almost 5 mm of anterior tibial translation [6]. Similar to current literature [3, 18, 19, 33], good-to-excellent objective knee stability was reported in the present study. When assessing a.p. instability clinically by performing the Lachman and Pivot-shift test, approximately 85% of patients in this cohort showed normal laxity.

Overall, outcomes scores such as Lysholm score and Tegner Activity Scale have been considered common outcome parameters of studies evaluating surgical ACL treatment. Comparable to the results of this study (88.9 ± 14.0), the Lysholm score outcomes were high in multiple studies after ARIF of tibial eminence fractures [3, 7, 10, 18, 31, 36, 45]. Similar to the results of Xu et al. [45], about 70% of patients reached excellent or good results in the Lysholm score in the present study. Since ACL-R results in high return-to-sport rates with clear limitations in terms of postoperative sports activity level [16, 20–22], physical activity remains an important outcome parameter after ARIF of tibial eminence fractures. To date, only Callanan et al. investigated on sport activities and return-to-sport following ARIF after tibial eminence avulsion fractures [5]. They indicated that postoperatively, their patient cohort returned to high impact sport activities, such as football, soccer, skiing, skating, and horse riding [5]. Of interest, Hirschmann et al. and Perugia and colleagues mentioned that preinjury sports activity level was reached by all patients without further specifications [11, 33]. Similarly [10, 11, 18], the median Tegner Activity Scale, which did not change postoperatively, was high in the present patient cohort, indicating no change in postoperative physical activity levels. Nevertheless, in the present study, two subjects claimed that physical activity deteriorated postoperatively. Another important finding was that return to high impact sport activities, such as skiing, cycling, hiking, and fitness was high after isolated ARIF of tibial eminence fractures (failures excluded). Subsequently, a high return-to-sports rate may be expected in this mostly young, active, and challenging patient cohort.

In consideration of the clinical and functional findings in this study, immediate refixation of the tibial eminence using the presented technique should be considered in these patients, as future ACL-R may be avoided. Additionally, when compared to adults, the outcome after ACL-R was demonstrated to be worse in children and adolescents [28, 35]. As such, the presented technique has multiple advantages when compared to conventional ACL-R: the minimal invasive approach allows for fast restoration of native biomechanical properties of the ACL in skeletally immature patients. Even if the reported revision and postoperative complication rate was noted to be high, this procedure does not destroy the path, even if the refixation of the unstable fragment fails. In addition, reposition of the fractured eminence fragment can be adjusted manually and individually to each patient, resulting in individual adaptation of tension and stability of the native ACL. Since mostly younger patients are affected of this type of injury, it may be of clinical relevance, as any muscular dysbalance due to harvesting of hamstring or quadriceps tendons may be avoided. Furthermore, it was recently reported that ACL-R in children and adolescents can lead to growth disturbances evolving throughout the entire process of remaining growth [29, 35].

Unfortunately, secondary ACL-R was required in two patients after ARIF in this cohort. This number is comparable to previous data published by Mitchell et al., who reported that 12% of patients required future ACL-R secondary to a delayed ACL rupture following ARIF of a tibial eminence fracture [26]. Possible explanations include elongation of ACL fibers at the moment of trauma followed by knee instability and a higher risk of ACL injuries secondarily [6, 37].

Interestingly, concomitant meniscal injuries occurred in eight patients (35%), which is similar to a recent study by Feucht et al., who demonstrated a 37% incidence of meniscal injury at the time of a tibial eminence fracture [8]. This high number of concomitant meniscal injuries in patients with tibial eminence avulsion fracture may occur secondary to higher-energy impaction of the tibia plateau against the femoral condyle during the high-energy pivoting injury mechanism. In addition, these finding could be attributable to anatomic proximity of the anterior root of the lateral meniscus as well as to a change in athletic activities with an increasing number of pivot-type injuries lately [8, 27]. However, similar to previous research after ACL-R [22], the results of the present study led to the assumption that additional meniscal lesions did not have an effect on postoperative stability or return-to-sport activity following ARIF.

When reviewing current literature, postoperative stiffness due to arthrofibrosis or mechanical impingement of displaced bony fragments is a common complication after ARIF of tibial eminence fractures [30–32, 43, 44]. According to a systematic review of Gans et al. [10], loss of ROM after tibial eminence fractures can be expected in up to 28%. Furthermore, the authors described existing knee laxity in up to 43% after ARIF [10]. Similar to their results, loss of ROM was evident in 29% of examined patients in the present study. However, only hyperextension was affected in this patient cohort, indicating that an extension of 0° could be achieved in all patients (extension/flexion 0°/0°/x°). In contrast to the results of Gans et al. [10], postoperative knee laxity was 100% normal or nearly normal.

The presented study has several limitations. First, since the retrospective study design inherently limits scientific objectivity and no control group was available, selection bias may have influenced the results. Second, the sample size was small, however, as these injuries remain a rare condition, the sample size is similar to previously published series. However, the statistical power of the present study was 0.9. Third, concomitant meniscal injuries were not excluded from this study may influencing the presented findings. However, according to previous research, meniscal lesions were shown to have no effect on return-to-sport activity following ACL-R [22]. Still, combined procedures may induce performance bias. Fourth, the pivot-shift test is subjective and depends on the experience of the investigator. Furthermore, the final average follow-up of 57 months might not be sufficient to evaluate the long-term functional and clinical outcomes after ARIF of tibial eminence fractures.

## Conclusion

Excellent Reliable ligamentous stability and high rates of return to high impact sports can be expected after ARIF using a suture fixation technique for type II–IV tibial eminence fractures. Complications, such as limitations in ROM, commonly occur in up to 30% after ARIF. Therefore, regular follow-up examinations remain important in this usually young patient cohort.
